# Application of Orange Peel Waste as Adsorbent for Methylene Blue and Cd^2+^ Simultaneous Remediation

**DOI:** 10.3390/molecules27165105

**Published:** 2022-08-11

**Authors:** Stephanie Giraldo, Nancy Y. Acelas, Raúl Ocampo-Pérez, Erika Padilla-Ortega, Elizabeth Flórez, Camilo A. Franco, Farid B. Cortés, Angélica Forgionny

**Affiliations:** 1 Grupo de Investigación Materiales Con Impacto (Mat&Mpac), Facultad de Ciencias Básicas, Universidad de Medellín, Carrera 87 No. 30-65, Medellin 050026, Colombia; 2 Centro de Investigación y Estudios de Posgrado, Facultad de Ciencias Químicas, Universidad Autónoma de San Luis Potosí, San Luis Potosi 78260, Mexico; 3 Grupo de Investigación Fenómenos de Superficie Michael-Polanyi, Facultad de Minas, Universidad Nacional de Colombia, Sede Medellín Kra 80 No. 65-223, Medellin 050034, Colombia

**Keywords:** agroindustrial waste, water treatment, adsorption process, cadmium, methylene blue, orange peel, multicomponent adsorption

## Abstract

Pollution by dyes and heavy metals is one of the main concerns at the environmental level due to their toxicity and inefficient elimination by traditional water treatment. Orange peel (OP) without any treatment was applied to effectively eliminate methylene blue (MB) and cadmium ions (Cd^2+^) in mono- and multicomponent systems. Although the single adsorption processes for MB and Cd^2+^ have been investigated, the effects and mechanisms of interactions among multicomponent systems are still unclear. Batch experiments showed that in monocomponent systems, the maximum adsorption capacities were 0.7824 mmol g^−1^ for MB and 0.2884 mmol g^−1^ for Cd^2+^, while in multicomponent systems (Cd^2+^ and MB), both contaminants competed for the adsorption sites on OP. Particularly, a synergic effect was observed since the adsorption capacity of Cd^2+^ increased compared to the monocomponent system. Results of desorption and adsorbent reuse confirmed that the adsorbent presents good regeneration performance. The low cost of this material and its capacity for the individual or simultaneous removal of Cd^2+^ and MB in aqueous solutions makes it a potential adsorbent for polluted water treatment processes.

## 1. Introduction

Nowadays, because of the rapid development of urbanization and industrialization worldwide, two environmental problems have been generated and require effective solutions. The first is water pollution by heavy metals and organic dyes caused by the continuous discharge of these contaminants from different industrial sectors such as the textile, printing, electroplating, mining, and metal etching industries [1,2,3]. It has caused damage to human health and aquatic ecosystems [4,5]. For instance, methylene blue (MB) is considered a hazardous organic contaminant as it is toxic, environmentally persistent, non-biodegradable, and resistant to solar degradation. Additionally, the release of MB into water sources represents an essential risk to aquatic life and animals further up the food chain, including humans [6].On the other hand, heavy metals such as cadmium are also very dangerous. Although this metal ion (Cd^2+^) has no pathological significance, it forms highly toxic compounds that can cause chronic poisoning in humans and animals, and long-term exposure to low concentrations of Cd^2+^ compounds can cause anemia, emphysema, neuralgia, stomach pain, and osteoporosis, while high Cd concentrations in the body affect enzymatic activities, the kidneys, bones, and respiratory systems [7].

The second environmental problem is the generation of high amounts of agroindustrial wastes that are directly discharged into landfills without any added use-value [8,9,10]. For instance, the world’s orange production was 49.6 million tons in 2016–2017. Orange peel accounts for around 50% of the total weight of the orange fruit. Hence, over 15.6 million tons of orange peels are discarded every year [11,12].

During the last decades, wastewater treatment for the removal of dyes and heavy metals has become a research area of growing interest. The use of adsorbent materials produced from agroindustrial wastes had made adsorption technology a sustainable treatment for the elimination of pollutants from wastewater [10,13,14,15]. Previous studies have reported good adsorption capacities for adsorbents produced from wood, fruit peels, rice husks, and seeds, among others [4,10,16,17,18,19,20,21] in the removal of heavy metals and dyes from wastewater.

Particularly, adsorbent materials derived from orange peel have shown high adsorption capacities for Cd^2+^ and MB [22,23,24,25,26,27]. Although these adsorbents have proved to be efficient, most available research studies are focused on adsorption in monocomponent systems. In addition, explanations of the involved adsorption mechanisms are limited. Therefore, understanding aspects such as adsorbate–adsorbent interactions, the simultaneous removal of both dyes and heavy metals, and the knowledge of the specific characteristics of each system can contribute to the design of more efficient adsorbents and improving the operating conditions in the adsorption process [28,29,30,31].

Previous reports have shown that the effluents of the textile and dyeing industries contain heavy metals, since these are used as a mordant agent in the dyeing process [32]. Hence, wastewater is a complex mixture of inorganic and organic compounds. The differences in the physicochemical properties of both pollutants make the treatment of wastewater a challenge, and the simultaneous removal of these pollutants has become a topic of interest [33]. In this sense, conducting studies in multicomponent systems is a necessary step to gain a better understanding of real systems [34,35,36]. Analysis of multicomponent systems allow establishing whether there is competition or synergy in the adsorption of any of the tested adsorbates [33,37]. In recent years, several studies have focused their research on the adsorption capacity of multicomponent systems for dyes and heavy metals [30,38,39,40]. For example, Li et al. [41] prepared an adsorbent material for the simultaneous removal of MB and Cu^2+^. Their results showed that the adsorption capacities for Cu^2+^ and MB gradually decreased in the binary system compared to their monocomponent systems, as the concentration of one or the other increased. On the other hand, Song et al., 2019 [33] synthesized an adsorbent from xanthate-modified baker’s yeast. Their findings indicated that the adsorption capacity for MB is hardly affected by the presence of Cd^2+^ in the binary solution (Cd^2+^ and MB). However, the adsorption capacity for Cd^2+^ increased in the presence of MB. The authors emphasize that this behavior occurs due to the chelation of metal ions with MB in response to electrostatic interactions between the adsorbent and the dye, which generate additional binding sites for the metal ions. Xiong et al., 2019 [42] evaluated the adsorption capacity of a magnesium biocomposite in a Cd^2+^–MB binary system. They found that Cd^2+^ adsorption capacity showed a slight increase in the multicomponent system compared to the monocomponent metal system, while a decrease of 18% in the MB adsorption capacity was observed because of the Cd^2+^ presence in the multicomponent system. This behavior is attributed to the direct competition between Cd^2+^ and MB for the same adsorption sites in the biocomposite, where interactions are mainly electrostatic. Moreover, some researchers have studied the adsorption of MB and Cd^2+^ in monocomponent systems using orange peel (OP) as adsorbent [22,24,25,26,27,43,44,45], but according to the author’s knowledge, there are no reports on simultaneous adsorption of both pollutants using OP without any transformation. Therefore, the aims of this work were: (i) to use OP waste as potential adsorbent for simultaneous Cd^2+^ and MB remediation, (ii) to evaluate the synergistic and/or antagonistic effect in the multicomponent adsorption process for these pollutants, determining the main interactions involved in each process. The adsorption experiments were carried out in monocomponent and multicomponent systems, using methylene blue (MB) and Cd^2+^. Isotherm parameters were determined to calculate the adsorption capacity and favorability of the adsorption process. Moreover, the surface of the adsorbent was characterized using FTIR to determine possible adsorbent–contaminant interactions.

## 2. Materials and Methods

### 2.1. Preparation and Characterization of the Adsorbent

The orange peel (OP) residue from a juice industry in the city of Medellín, Colombia was used as the adsorbent. It was washed several times with distilled water to remove impurities. Then, the wet biomass was dried in an oven at 105 °C for 12 h. Dry biomass was cut into small pieces and ground until yielding a particle size of less than 0.420 mm. Finally, the dry biomass was stored until use.

The functional groups in the adsorbent and their interactions with MB and Cd^2+^ were determined by using Fourier transform infrared spectroscopy (FTIR) (Spectrum two-PerkinElmer with UATR), in the range from 4000 to 450 cm^−1^, before and after the adsorption process.

### 2.2. Adsorption Experiments

Adsorption experiments using OP as an adsorbent were performed in batch mode with a solution pH value higher than the MB pKa (pKa = 3.8) [46] to guarantee having MB positively charged. A solution of NaOH and HCl were prepared, keeping a 0.01 M constant ionic strength. On the other hand, according to the speciation diagram of cadmium [47], in aqueous solutions this metal can be found as Cd^2+^ (ionic form) up to pH 8. Therefore, adsorption tests for both contaminants were carried out at pH 7, ensuring that the positively charged species of these contaminants (MB^+^ and Cd^2+^) were available in solution. Previous adsorption studies have chosen this pH value for testing both pollutants [33,48,49].

### 2.3. Optimal Contact Time and Isotherms in Monocomponent Systems

Batch experiments were performed, mixing 50 mg of OP and 50 mL of MB or Cd^2+^ solution in 250 mL Erlenmeyer flasks. The initial concentration of each contaminant solution was 250 mg L^−1^ (2.22 mmol L^−1^ of Cd^2+^ and 0.78 mmol L^−1^ of MB). The pH of solutions was kept at 7 by the addition of a few drops of 0.1 M NaOH or 0.1 M HCl, depending on the need. Samples were shaken at 200 rpm, and the contact time was evaluated for periods of 6, 12, 24, 48, and 72 h. From these experiments, the optimal contact time was determined. All measurements of the residual contaminant concentration in solution were performed in a HACH spectrophotometer (VIS-DR 3900). MB concentration was measured at 665 nm using a calibration curve in the range of 0.0002–2.00 mmol L^−1^. On the other hand, the Cd^2+^ concentration was determined by colorimetry with the HACH 10217 (TNT-plus 852) method at 552 nm.

The adsorption capacity for MB and Cd^2+^ was determined with Equation (1):

(1) $$q_{t}=\frac{C_{0}-C_{t}}{w}\times V$$

where, q_t_ (mmol g^−1^) is the amount of the pollutant adsorbed at time t, C_0_ (mmol L^−1^) is the initial concentration of the pollutant in the solution, C_t_ (mmol L^−1^) is the concentration of the pollutant at time t, V (L) is the volume of the solution, and w (g) is the mass of the adsorbent material.

Adsorption isotherms were obtained for each pollutant, varying the concentrations from 50 to 250 mg L^−1^ (0.16–0.78 mmol L^−1^ for MB and 0.44–2.22 mmol L^−1^ for Cd^2+^), at the optimum contact time established (24 h). Subsequently, the data were fitted to the Langmuir [50], and Freundlich [51] isotherm models, according to Equations (2) and (3), respectively (see Table 1).

### 2.4. Adsorption in Multicomponent Systems

Adsorption in multicomponent systems of MB and Cd^2+^ on OP material was carried out under the experimental conditions mentioned above. A 3 × 3 matrix was defined by combining the concentrations of 50, 100, and 200 mg L^−1^ for both contaminants. That was 0.16, 0.31, 0.63 mmol L^−1^ for MB and 0.44, 0.89, 1.78 mmol L^−1^ for Cd^2+^.

The ratio of the adsorption capacities (R_q_) expressed by Equation (4) helps to understand the effect of each contaminant in multicomponent systems.

(4) $$R_{q}=\frac{q_{e,i}}{q_{0,i}}\;$$

where q_e_ and q_0_ are the adsorption capacities of the pollutant *I* in the binary system and the monocomponent system, respectively. Previous studies have reported that if R_q_ > 1, the adsorption of pollutant *I* was promoted by the presence of the other pollutant; if R_q_ = 1, there was no effect on the adsorption capacity for the pollutant *I*; if R_q_ < 1, the adsorption of pollutant *I* was inhibited by the presence of the other pollutant. It is useful to determine the effect of both pollutants on the adsorbent performance when a binary system is studied [33].

### 2.5. Adsorbent Reusability

To study desorption and reuse cycles of the adsorbent, 0.2 g of OP was added in 200 mL of a combined MB-Cd^2+^ solution (200 mg L^−1^ of each contaminant: 0.63 mmol L^−1^ for MB and 1.78 mmol L^−1^ for Cd^2+^), and it was shaken for 24 h. After saturation, the adsorbent was filtered, and then it was submitted to the desorption process. For desorption, a mixture of 20 mL of a 1% HCl solution in ethanol (*v*/*v*), and 20 mL of HNO_3_ (1 M) was placed in contact with the adsorbent for 5 h. Finally, the adsorbent was washed with deionized water, dried for 12 h in an oven at 105 °C, then stored until the next adsorption cycle.

## 3. Results and Discussion

### 3.1. Adsorption Isotherms in Monocomponent Systems

**Figure 1:**
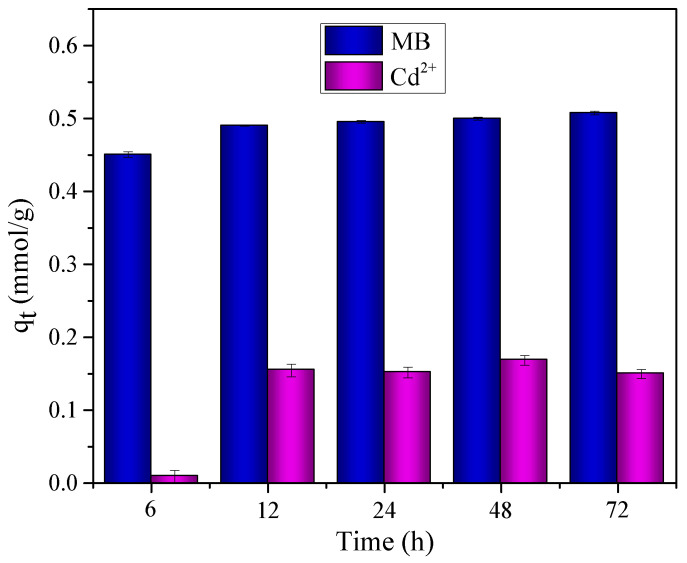
Optimum contact time for MB and Cd^2+^ on the adsorbent material OP.

Figure 1 shows a fast adsorption during the first 6 h for MB; the adsorption capacity obtained at this time was 0.45 mmol g^−1^, while Cd^2+^ was adsorbed in low quantity (0.009 mmol g^−1^) during the first hours of the process. However, both pollutants reached equilibrium at 24 h, with adsorption capacities of 0.50 mmol g^−1^ for MB and 0.14 mmol g^−1^ for Cd^2+^; the adsorption capacities obtained for times greater than 24 h were kept constant. This time was chosen in order to obtain the adsorption equilibrium.

**Figure 2:**
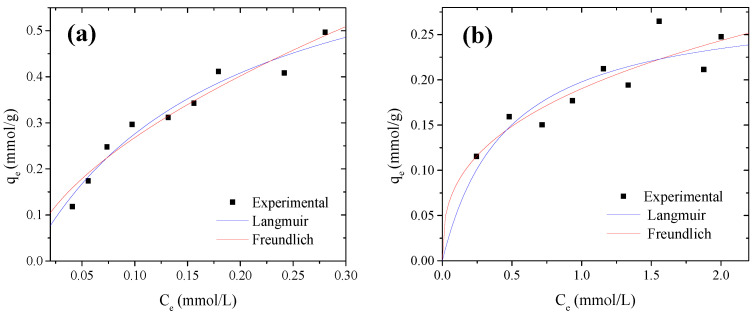
Langmuir and Freundlich isotherms for (**a**) MB and (**b**) Cd^2+^.

Adsorption isotherms are very helpful models for describing how solutes interact with adsorbents and allow one to calculate the maximum adsorption capacity of the materials. Monolayer adsorption is assumed by the Langmuir isotherm [52], where the surface has a finite number of active sites for adsorbate adsorption and the system is homogeneous. However, the Freundlich model assumes a heterogeneous adsorbent surface with multilayer adsorption, where the strongest binding sites are occupied first [51]. Table 2 shows the isotherm parameters found for MB and Cd^2+^. A good fit of the data was observed for both models comparing R^2^ values, which were close to 0.9 (see Figure 2). However, the root-mean-squared error (RMSE) and sum of square errors (SSE) were calculated to validate which of the two isotherms best fit the adsorption of MB and Cd^2+^. From these values (see Table 2), it can be observed that the experimental data fit the Langmuir isotherm better than Freundlich for MB, while for Cd^2+^, the RMSE values were very close (0.021 and 0.020), suggesting that both isotherm models adjusted well the adsorption data. In the Freundlich model, the value of ‘n’ is indicative of the adsorption favorability. Values of ‘n’ in the range from 2 to 10 suggested good, 1–2, quite difficult, and less than 1, poor adsorption characteristics. For Cd^2+^, the obtained *n* value was >2, indicating that adsorption is a favorable physical process [53], with multilayer adsorption on heterogeneous active sites of OP surface (Freundlich-type) [54]. Although an *n* < 2 for MB suggested difficult adsorption for MB, the RMSE indicated that the Langmuir model was better adjusted for MB adsorption (0.023 vs. 0.027), assuming that monolayer adsorption was preferred for MB. Additionally, the higher value of K_L_ for MB than for Cd^2+^ indicated its major affinity towards the adsorbent, suggesting that the adsorbent–MB interactions were favored.

The maximum adsorption capacities $\left(Q_{m}\right)$ were 0.7824 mmol g^−1^ (250.6 mg g^−1^) and 0.2884 mmol g^−1^ (32.4 mg g^−1^) for MB and Cd^2+^, respectively. The difference in adsorption capacity for both contaminants may be due to the physicochemical properties of the adsorbent material and the adsorbates. Depending on these specific characteristics, the adsorption process can be favored. The obtained capacities were similar to or higher than those achieved in other studies using orange peel without any treatment. For example, maximum adsorption capacities of 14.16 and 218 mg g^−1^ for MB [22,44] and 35.71 mg g^−1^ for Cd^2+^ [23] have been reported. For comparison proposes, Table 3 presents the maximum adsorption capacity obtained for MB and Cd^2+^ using OP without any treatment vs. adsorption capacities using different adsorbents obtained by transformation of OP.

Moreover, the selectivity ratio, calculated as S = (qMB/qCd^2+^) from the isotherm parameters, showed that OP had an MB adsorption capacity 7.7 times higher than that for Cd^2+^ at 25 °C and pH = 7, suggesting that OP has a higher affinity for the MB molecules in monocomponent systems. Thus, the good adjustment of data to both isotherm models suggested that there is a mixture of adsorption mechanisms. Therefore, the pollutants are adsorbed on the OP through different interaction energies, which are determined by their functional groups.

**Figure 3:**
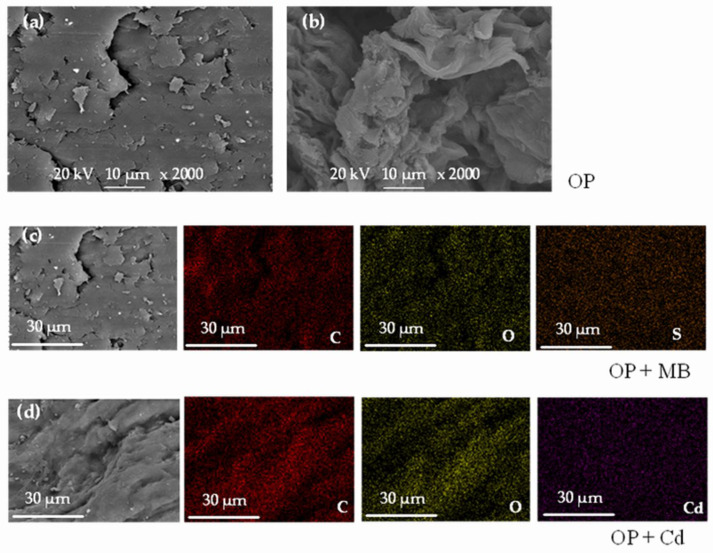
SEM-EDS elemental mapping of OP after MB and Cd^2+^ adsorption in monocomponent systems. The element distributions were represented by colors red (carbon), yellow (oxygen), orange (sulfur), and violet (cadmium). (**a**,**b**) SEM images of OP at ×2000 magnification. (**c**) SEM-EDS images of OP sample after MB adsorption, and (**d**) SEM-EDS images of OP sample after Cd^2+^ adsorption.

The SEM images of the OP sample are shown in Figure 3 after adsorption in monocomponent systems for both pollutants. From the micrographs, a heterogeneous structure of OP is observed, with laminar structure zones and agglomerates in other zones (Figure 3a,b). From EDS analysis, a wide distribution of S (which is present in the MB molecule) and Cd can be observed on the adsorbent surface, indicating that the adsorption of the dye and the metal ion took place in monocomponent systems (Figure 3c,d). Other metals such as K, Ca, Na and Mg were also observed in the elemental analysis by EDS. The elemental mapping for the three samples (OP, OP + MB, and OP + Cd) is displayed in Figure S1 in the supplementary material.

### 3.2. Effect of Pollutant Equilibrium Concentration in Multicomponent Systems

**Figure 4:**
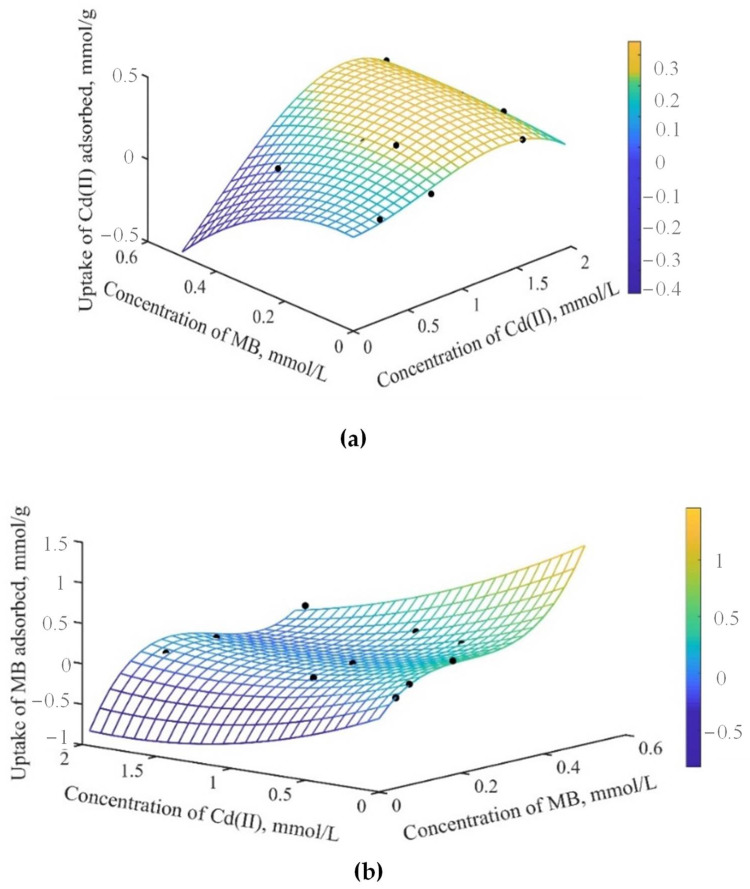
Effect of the equilibrium concentration of pollutants in multicomponent systems. (**a**) Adsorption capacity for Cd^2+^ in the presence of MB. (**b**) Adsorption capacity for MB in the presence of Cd^2+^.

**Figure 5:**
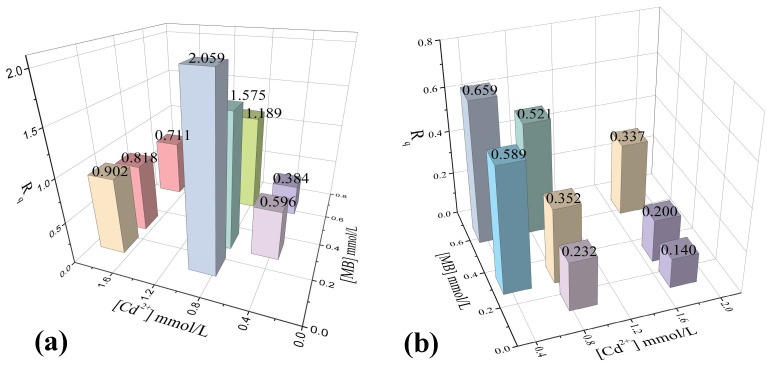
Relationship of the adsorption capacity (Rq) at initial concentrations of both contaminants. (**a**) Rq for Cd^2+^ in the presence of MB. (**b**) Rq for MB in the presence of Cd^2+^.

The multicomponent adsorption equilibrium data for MB and Cd^2+^ on OP are represented in Figure 4. Figure 4a shows the effect of MB presence (concentration range: 0.04–0.51 mmol L^−1^) on the capacity of OP to adsorb Cd^2+^. Two trends were observed depending on the equilibrium concentration of both pollutants. At low equilibrium concentrations of Cd^2+^ (0.34 and 0.45 mmol L^−1^), cadmium adsorption capacities decreased by 40 and 62%, respectively, compared to that on the monocomponent system, i.e., this effect was more evident with the increase in the concentration of MB. Thus, these results suggest that there is a more competitive effect between both pollutants. In binary systems of Cu^2+^ and MB, a competitive effect as the concentration of one or the other contaminant increases has also been reported [41]. Similar behavior was observed at the highest concentrations of Cd^2+^ (1.8–1.9 mmol L^−1^), since the adsorption capacity of Cd^2+^ decreased as the concentration of MB increased (0–0.51 mmol L^−1^). Nevertheless, the competitive effect observed between the two pollutants was less intense at high concentrations of Cd^2+^. The adsorption capacity for Cd^2+^ only decreased from 10 to 28% (compared to monocomponent systems) as the MB concentration rose to 0.51 mmol L^−1^. Hence, the contaminants competed for the same active sites available on OP. Moreover, since the pH of the solution (pH = 7) was higher than the pH_PZC_ (3.5) of OP, the interactions that governed the adsorption process were electrostatic. However, cationic dye molecules are larger than Cd^2+^ cations and thus have greater contact surfaces. Therefore, they could interact with the adsorbent and block some active sites for Cd^2+^ adsorption. This inhibition of Cd^2+^ adsorption generated by the presence of MB was evidenced by the values of R_q_ < 1 for these two systems (see Figure 5a).

At medium equilibrium concentrations of Cd^2+^ (0.80–0.92 mmol L^−1^), the Cd^2+^ adsorption capacities increased from 30 to 50% compared to the monocomponent system as the MB concentration rose to 0.51 mmol L^−1^. This indicates that there was a synergistic effect on Cd^2+^ adsorption, which was corroborated with the values of R_q_ > 1 (see Figure 5a). Song et al. [33] studied the simultaneous adsorption of MB and Cd^2+^, finding that the adsorption capacities of the metal were improved by the presence of the dye. The authors proposed that the formation of metal–dye complexes provides additional binding sites for the metal. In this sense, our results suggest that some Cd^2+^ ions are adsorbed directly on OP surface, and the other ones are removed by complexation with MB molecules previously adsorbed [28].

On the other hand, the effect of Cd^2+^ concentration (0–1.9 mmol L^−1^) on the adsorption capacity of OP to adsorb MB in the multicomponent system is observed in Figure 4b. Results indicate that the adsorbed amount of MB gradually decreased in the multicomponent system, being more evident at low concentrations of MB. For instance, the adsorption capacity for MB in the multicomponent system decreased between 41 and 86% compared to the monocomponent system at equilibrium concentrations of MB (0.13–0.27 mmol L^−1^). However, at the highest equilibrium concentrations of MB (0.36–0.51 mmol L^−1^), a decrease from 34 to 66% in the adsorption capacity for MB was observed due to the presence of Cd^2+^ in the solution. Then, the elimination of MB was strongly affected by the presence of Cd^2+^. Moreover, these results confirm that the adsorption of MB and Cd^2+^ in multicomponent systems is competitive, since both contaminants can interact with the same active sites of OP by electrostatic attraction. Because of the small size of Cd^2+^ ions and the higher concentration of Cd^2+^ than MB in all multicomponent systems, higher mobility of Cd^2+^ ions could be expected in solution. Thereby, metal ions could rapidly interact with the active sites in OP, reducing the number of active sites available for MB adsorption [28]. Furthermore, the R_q_ values were <1 (see Figure 5b), indicating that MB adsorption was inhibited by the presence of Cd^2+^. Xiong et al. [28] studied the adsorption of Cd^2+^ and MB on zeolites in multicomponent systems. Their results were like those mentioned above, concluding that MB adsorption decreases because of the direct competition between Cd^2+^ and MB for the same adsorption sites. Additionally, the authors established that the Cd^2+^ is preferentially adsorbed on the adsorbent surface. Huang et al. [29] studied the simultaneous adsorption of MB and Cu^2+^ onto citric acid cross-linked β-cyclodextrin, finding that the adsorption of either pollutant was affected by the presence of the other. The observed competitive effect was associated with the interaction of the two pollutants with carboxylate groups (-COO^−^) on the surface of the material, forming complexes with Cu^2+^ and interacting with MB through electrostatic attraction.

The analysis of the variation in the adsorption capacities for both pollutants as a function of their equilibrium concentrations revealed a competitive effect between Cd^2+^ and MB for the adsorption sites on OP. These results suggested that the adsorption process is dominated by electrostatic-type attractions between the surface functional groups of the adsorbent and the contaminants in most concentrations. However, a synergistic effect between both pollutants was particularly observed at equilibrium concentrations from 0.80 to 0.92 mmol L^−1^, which was responsible for the increase in Cd^2+^ adsorption.

It can be highlighted that the different behavior and adsorption capacities of adsorbent materials reported in the literature in multicomponent systems of Cd^2+^ and MB and the adsorbent material used in this work could be associated with their chemical nature. Hence, the study of adsorbent surface functional groups and the identification of different contaminant–adsorbent interactions are essential to achieve a better understanding of all the phenomena taking place in multicomponent systems.

### 3.3. Effect of Pollutant Equilibrium Concentration in Multicomponent Systems

**Figure 6:**
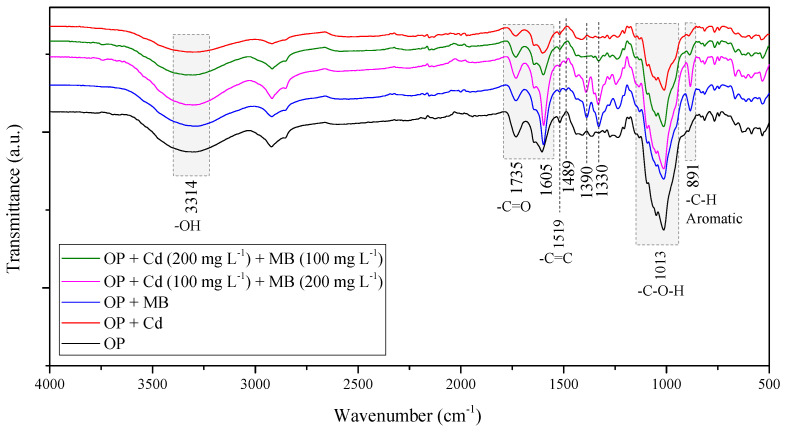
FTIR spectra of the OP before and after adsorption.

**Figure 7:**
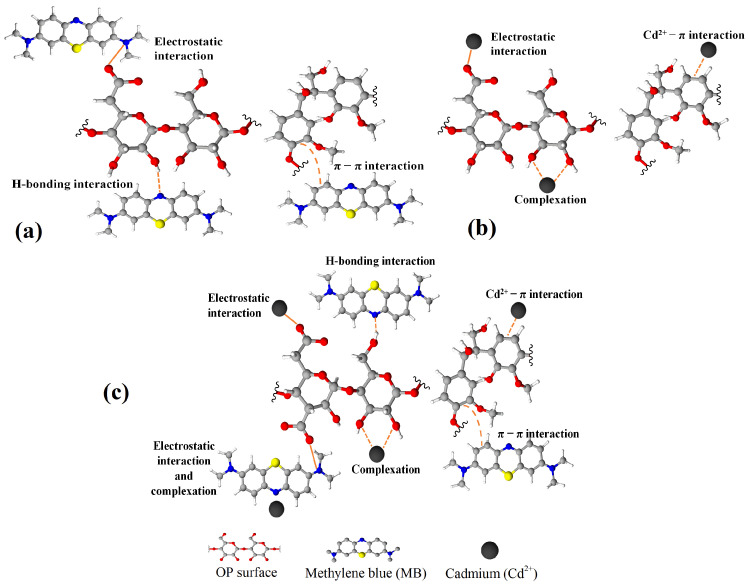
Schematic representation of the adsorption mechanisms on OP surface. (**a**) Interaction with MB. (**b**) Interaction with Cd^2+^. (**c**) Interactions with binary systems of MB and Cd^2+^.

The characterization of OP was previously reported by our research group [31]. A BET surface area of 3.098 m^2^/g was determined for this material, suggesting that the adsorption process will be dominated by favoring contaminant–adsorbent interactions. FTIR analysis was performed before and after the adsorption process (see Figure 6, Table 4) to identify the main interactions of Cd^2+^ and MB with the OP surface. After adsorption of MB, the FTIR spectrum of OP (OP + MB) showed a shift to lower wavenumbers (3314 cm^−1^ vs. 3296 cm^−1^) of the band associated with -OH stretching vibrations and a new peak appeared around 1390 cm^−1^. Both changes were associated with the existence of hydrogen bonds between hydroxyl groups of OP surface and the N atom of MB [29]. The band corresponding to the symmetric deformation of the -CH_3_ group of the MB molecule was slightly redshifted from 1330 cm^−1^ to 1329 cm^−1^. In addition, a characteristic band of MB associated with the -C=N group vibrations showed a shift from 1605 to 1598 cm^−1^. Previous studies have reported that the shifts in the FTIR spectra bands are related to changes in the strength of the chemical bond. Hence, an FTIR band shift to lower wavenumbers indicates a weakening of the chemical bond, while a shift to higher wavenumbers indicates that the chemical bond was strengthened [57]. On the other hand, a new band associated with group C=C vibrations was observed at 1489 cm^−1^, and the band related to aromatic -C-H vibrations was shifted from 891 to 882 cm^−1^. These two findings suggest that π–π interactions are involved in the adsorption process; the π–π interactions occurred between the aromatic structure of the adsorbent and the benzene-type rings in the MB molecule [41]. In addition, a redshift (1734 cm^−1^ vs. 1732 cm^−1^) and a decrease in the intensity of the band associated with the vibrations of the -C=O indicate the occurrence of electrostatic interactions between the positively charged N atom of the MB molecule and the carboxylate (-COO^−^) groups on the surface of OP (see Figure 7a) [58,59,60]. In the FTIR spectrum of OP after the adsorption of Cd^2+^ (OP + Cd), it was observed that the stretching vibration of the -O–H band (3314 cm^−1^) was shifted to 3290 cm^−1^, indicating the presence of interactions between Cd^2+^ and the hydroxyl groups of OP. Moreover, a decrease in the intensity of the band at 1735 cm^−1^ associated with the vibrations of the -C=O group was observed, and the band at 1013 cm^−1^ associated with the -C-O-H vibrations shifted to 1010 cm^−1^. According to the literature, the redshifts and the decrease in the intensity of the FTIR bands have been associated with the complexation of metal ions with O atoms in the adsorbent, which can share their lone pairs of electrons to coordinate metal ions [61,62,63]. Furthermore, the occurrence of these interactions has been confirmed by other studies, which concluded that carboxyl and hydroxyl groups are the main functional groups involved in the mechanisms during the adsorption process of metal ions [19,42,64]. Two additional redshifts were observed for the bands associated with the stretching of the aromatic double bond C=C (1519 cm^−1^) and the stretching of the aromatic -C-H bond (891 cm^−1^). After Cd^2+^ adsorption, the FTIR bands were located at 1517 cm^−1^ and 889 cm^−1^, respectively [37,65]. Previous studies have attributed these band shifts to the establishment of interactions between Cd^2+^ and the π electrons of the aromatic structure [62,66]. A schematic representation of the Cd^2+^ adsorption process on OP is presented in Figure 7b. The selectivity ratio (S) calculated for the contaminants in monocomponent systems showed a higher affinity of OP towards MB than Cd^2+^. Previous reports [41] have proposed that the MB adsorption preference versus the metal ions could be attributed to the main interactions governing the MB adsorption process, i.e., the π-π interactions, hydrogen bonding, and electrostatic attraction, whereas the Cd^2+^ adsorption process is governed by complexation interactions.

Looking closely at the spectra after adsorption of both contaminants, minimal variations were observed compared to the FTIR spectra of OP in monocomponent systems. However, it was observed that the bands of the OP spectra after the adsorption of MB and Cd^2+^ were more intense in the spectrum of the multicomponent system with initial concentrations of MB and Cd^2+^ of 200 mg L^−1^ and 100 mg L^−1^, respectively. Furthermore, the OP + Cd100 + MB200 spectrum was similar to the OP + MB spectrum. For these concentrations, the amount of MB adsorbed in the equilibrium (*q_MB_* = 0.21 mmol g^−1^) was higher than the amount of Cd^2+^ adsorbed (*q_Cd_* = 0.18 mmol g^−1^). This fact was also corroborated by the equilibrium concentrations of both pollutants, whose values showed that the equilibrium concentration of MB (*C_e, MB_* = 0.42 mmol L^−1^) was lower than that of Cd^2+^ (*C_e, Cd_* = 0.92 mmol L^−1^) in the solution. As discussed in Section 3.2, under these conditions, there is a synergistic effect on Cd^2+^ adsorption due to the generation of new adsorption sites for the metal ion by complexation with the MB molecule. Although the FTIR spectra do not provide information about this possible complexation, Song et al. [33] evidenced this phenomenon in their work. Contrarily, the FTIR spectrum of OP after adsorption of both pollutants using initial concentrations of MB of 100 mg L^−1^ and Cd^2+^ of 200 mg L^−1^ (OP + Cd200 + MB100) showed that the FTIR bands were similar to the spectrum of the monocomponent system of Cd^2+^. This occurred because, for this combination of concentrations, the adsorption capacity for Cd^2+^ (*q_Cd_* = 0.22 mmol g^−1^) was higher than that for MB (*q_MB_* = 0.05 mmol g^−1^), which is corroborated by their equilibrium concentrations (*C_e, Cd_* = 0.27 mmol L^−1^ and *C_e, MB_* = 1.84 mmol L^−1^).

Analyzing all of the results obtained in the multicomponent system and the FTIR spectra, it can be established that there is competition between both contaminants for the active sites of OP. Additionally, it was deduced that the metal ion has higher mobility than MB in aqueous solution because of its smaller size and higher availability in solution for the studied multicomponent system. Particularly, a synergistic effect was found in the mechanism for adsorption of Cd^2+^ and MB at equilibrium concentrations of Cd^2+^ between 0.80 and 0.92 mmol L^−1^, since the values of the adsorption capacity for Cd^2+^ were higher than those in the monocomponent system. In our previous study [31], OP was characterized by Boehm titration, and a high concentration of oxygenated functional groups was determined (carboxylic, 0.650 mmol g^−1^; lactone, 0.925 mmol g^−1^; and phenol, 0.200 mmol g^−1^) on the surface of OP. It has been reported that the presence of these active sites in the adsorbent contributes to obtaining good adsorption capacities for the removal of metal ions and dyes. In addition, as the point of zero charge pH_(PZC)_ of OP was 3.5, the electrostatic interactions with cationic pollutants were favored under working conditions (pH = 7). Therefore, the MB and Cd^2+^ adsorption process was governed by the occurrence of different interactions between both pollutants and the adsorbent, such as: *i.* electrostatic attraction between the pollutants and the OP surface, *ii.* cadmium complexation with the surface oxygenated groups of OP, *iii.* hydrogen bonds between hydroxyl groups of OP and MB, *iv.* π–π interactions (OP-MB) and π–electron interactions of OP-Cd^2+^, and *v.* complexation between Cd^2+^ and the MB molecule (See Figure 7c) [33,42].

Table 5 displays adsorption capacities in mono- and multicomponent systems to compare our adsorbent material with other adsorbent materials used for the removal of MB and Cd^2+^. From the table, it can be observed that antagonistic and synergistic effects took place in the adsorption process for multicomponent systems of both contaminants.

### 3.4. Reusability of the Adsorbent

**Figure 8:**
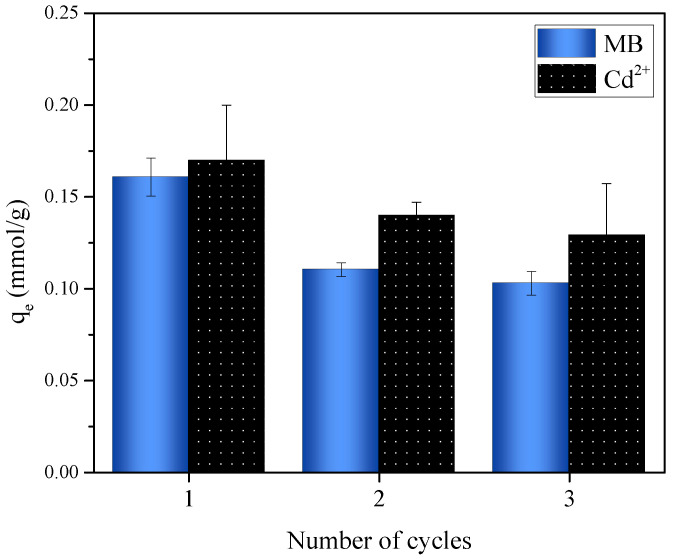
Regeneration of OP for the adsorption of MB and Cd^2+^ in a multicomponent system.

Adsorbent regeneration is a very important process since if an adsorbent can be reused in different adsorption cycles, the costs associated with the process are reduced. Figure 8 shows three reuse cycles performed with the OP material to evaluate the efficiency of the adsorbent after the desorption process. The adsorption capacity of OP to adsorb Cd^2+^ did not change drastically; that is, a decrease of 18% was observed for the second cycle and of 5% for the third cycle compared to its initial adsorption capacity. For MB, a decrease of 30% in adsorption capacity was observed for the second adsorption cycle compared to the first cycle, while for the third cycle a decrease of only 7% was observed compared to the second cycle. These results indicate that OP is a good option for practical applications since it can be used for several adsorption cycles without a significant loss of its adsorption capacity for the elimination of different contaminants present in aqueous solutions.

## 4. Conclusions

Orange peel without any treatment was efficient for the removal of MB and Cd^2+.^ For the monocomponent system, the fitting of the data to the Langmuir model allowed determining a maximum adsorption capacity of 0.7824 mmol g^−1^ for MB and of 0.2884 mmol g^−1^ for Cd^2+^. Adsorption results in the MB and Cd^2+^ multicomponent systems showed that the elimination of MB was strongly affected by the presence of Cd^2+^, which indicates that there is competition between both contaminants, since both contaminants can interact with the same active sites of OP. Particularly, the adsorption of both pollutants at equilibrium concentrations of Cd^2+^ in the range of 0.80–0.92 mmol L^−1^ led to a synergistic effect on the adsorption of Cd^2+.^ Hence, its adsorption increased compared to that observed in the monocomponent system, due to MB providing new active sites for the elimination of Cd^2+^ by complexation. From FTIR characterization, it was possible to identify the different interactions involved during the adsorption process in the multicomponent system. For systems with the synergistic effect on Cd^2+^ adsorption, an electrostatic interaction between the MB and the negatively charged surface of OP occurred together with the Cd^2+^ complexation by the MB molecule. Additionally, Cd^2+^ established interactions directly with the surface of OP through electrostatic attractions, complexation with surface functional groups of the adsorbent, and Cd^2+^–π interactions. Different interactions were also proposed for MB and OP. On the other hand, the results obtained after the desorption process show no significant effect on the adsorption capacity of OP for both pollutants between the second and third adsorption cycle. Hence, OP presents the necessary characteristics for the elimination of contaminants such as dyes and heavy metals commonly present in aqueous solutions.

## Figures and Tables

**Table 1:** Isotherm model equations.

Langmuir	$q_{e}=\frac{C_{e}Q_{m}K_{L}}{C_{e}K_{L}+1}\;\;\;\left(2\right)$
Freundlich	$q_{e}K_{F}C_{e}{}^{\frac{1}{n}}\;\;\;\left(3\right)$

C_e_: adsorbate concentration at equilibrium (mmol L^−1^); q_e_: amount of adsorbate adsorbed at equilibrium (mmol g^−1^); Q_m_: maximum capacity of adsorbate (mmol g^−1^); K_L_: constant of Langmuir (L g^−1^); K_F_: Freundlich dissociation constant (mmol g^−1^); n: constant related to the intensity of the reaction; R_L_: non-dimensional separation factor; C_i_: the initial concentration of adsorbate (mmol L^−1^).

**Table 2:** Isotherm parameters for MB and Cd^2+^ adsorption on OP.

Adsorbent	MB	Cd^2+^
**Langmuir**		
Q_m_ (mmol g^−1^, mg g^−1^)	0.7824, 250.6	0.2884, 32.4
K_L_ (L mmol^−1^)	5.44	2.18
R^2^	0.98	0.89
RMSE	0.023	0.021
SSE	0.005	0.004
**Freundlich**		
K_F_ (mmol g^−1^)	1.029	0.19
n	1.71	2.81
R^2^	0.97	0.90
RMSE	0.027	0.020
SSE	0.006	0.003

**Table 3:** Comparison of maximum adsorption capacities for MB y Cd^2+^ monocomponent system.

Treatment	Contaminant	Q_m_ (mg g^−1^)	pH	Adsorption Isotherm	Adsorption Mechanisms	Reference
OP washed	MB	250.58	7	Langmuir, Freundlich	Electrostatic interaction, H-bonding interaction, π–π interactions	This study
OP thermochemical activation using ZnCl_2_ (800 °C in N_2_ atmosphere)	MB	339.82	8	Sips y Langmuir	Electrostatic interaction, H-bonding interaction, π–π interactions	[16]
OP washed	MB	192.31	4.5	Langmuir, Freundlich, Temkin	Electrostatic interaction, H-bonding interaction, π–π interactions	[31]
OP washed (OP) and chemical activation using NaOH (SOP)	MB	14.16 OP 18.28 SOP	OP 4 SOP 9	Freundlich	Electrostatic attraction	[44]
OP chemical activation using H_3_PO_4_	MB	307.63	6.2	Langmuir	Electrostatic interaction, π–π interactions	[45]
OP washed	Cd^2+^	32.42	7	Langmuir, Freundlich	Electrostatic interaction, complexation, Cd-π interactions	This study
OP washed	Cd^2+^	59.5	7	Langmuir, Freundlich	Electrostatic interaction, complexation	[25]
OP washed and pyrolyzed at 700 °C	Cd^2+^	114.69	7	Langmuir	Cd-π interactions, surface precipitation	[55]
OP Washed	Cd^2+^	4.90	5	Langmuir	Not reported	[56]
OP modified with Fe_2_O_3_	Cd^2+^	76.92	7	Langmuir	Complexation, ion exchange	[23]

**Table 4:** FTIR bands before and after adsorption.

OP Bands (cm^−1^)	Bands after Adsorption (cm^−1^)				Functional Group
	OP-MB	OP-Cd^2+^	OP-Cd (100 mg L^−1^)-MB (200 mg L^−1^)	OP-Cd (200 mg L^−1^)-MB (100 mg L^−1^)	
3314	3296	3290	3305	3308	-OH
1734	1732	1734	1732	1732	-C=O
1605	1598	1602	1597	1599	C=N
1519	1517	1517	1517	1517	C=C
1330	1329	1326	1331	1329	-CH_3_
1013	1012	1010	1012	1012	C-O-H
891	882	889	882	886	C-H aromatic

**Table 5:** Comparison of maximum adsorption capacities of MB and Cd^2+^ multicomponent systems.

Adsorbent	Monocomponent Adsorption Q_m_ (mg g^−1^)		Multicomponent Adsorption Q_m_ (mg g^−1^)		Effect	Reference
OP washed	Cd^2+^ 32.42	MB 250.58	Cd^2+^ 34.8	MB 86.79	Synergistic (Cd^2+^ adsorption capacity increases in the presence of MB). Antagonistic (MB adsorption capacity is affected by the presence of Cd^2+^).	This study
Magnesium silicate biocomposite and subsequent hydrothermal carbonization	Cd (II) 104	MB 325	Cd (II) 108	MB 200	Synergistic (Cd (II) adsorption capacity showed a slight increase in the presence of MB). Antagonistic (MB adsorption capacity was affected by the presence of Cd (II) in the multicomponent system).	[42]
Xanthate-modified baker’s yeast	Cd^2+^ 224.5	MB 56.25	Cd^2+^ 267	MB 57.88	Synergistic (Cd^2+^ adsorption capacity increases in the presence of MB). MB adsorption capacity is almost unaffected by the presence of Cd^2+^.	[33]
Albizia lebbeck pods carbon (ALPC)	Cd^2+^ 185.19	MB 80.01	Cd^2+^ 181.8	MB 91.74	Antagonistic effect	[67]

## Supplementary Materials

The following are available online at https://www.mdpi.com/article/10.3390/molecules27165105/s1, Figure S1: SEM-EDS elemental mapping of OP after MB and Cd2+ adsorption in monocomponent systems.
