# Supplementary material for: Application of Orange Peel Waste as Adsorbent for Methylene Blue and Cd2+ Simultaneous Remediation
Source: Molecules. 2022 Aug 11;27(16):5105. doi: 10.3390/molecules27165105 (PMC9416566; doi:10.3390/molecules27165105)
Supplement: Supplementary file 1 [file molecules-27-05105-s001.zip › molecules-1818245-supplementary.pdf]

Supplementary Material

Application of Orange Peel Waste as Adsorbent for Methylene Blue and Cd^{2+} Simultaneous Remediation

Stephanie Giraldo [1], Nancy Y. Acelas [1,*], Raúl Ocampo-Pérez [2], Erika Padilla-Ortega [2], Elizabeth Flórez [1,*], Camilo A. Franco [3], Farid B. Cortés [3] and Angélica Forgionny [1,*]

[1] Grupo de Investigación Materiales Con Impacto (Mat&Mpac), Facultad de Ciencias Básicas, Universidad de Medellín, Carrera 87 No. 30-65, Medellin 050026, Colombia; stepha930925@hotmail.com (S.G.)
[2] Centro de Investigación y Estudios de Posgrado, Facultad de Ciencias Químicas, Universidad Autónoma de San Luis Potosí, San Luis Potosi 78260, Mexico; raul.ocampo@uaslp.mx (R.O.-P.); erika.padilla@uaslp.mx (E.P.-O.)
[3] Grupo de Investigación Fenómenos de Superficie Michael-Polanyi, Facultad de Minas, Universidad Nacional de Colombia, Sede Medellín Kra 80 No. 65-223, Medellin 050034, Colombia; caafrancoar@unal.edu.co (C.A.F.); fbcortes@unal.edu.co (F.B.C.)
* Correspondence: nyacelas@udemedellin.edu.co (N.A.); elflorez@udemedellin.edu.co (E.F.); mforgionny@udemedellin.edu.co (A.F.)

Results and Discussion

1. Additional Information of SEM Characterization

The SEM images and elemental mapping of the OP, OP+Cd, and OP+MB samples are shown in Fig. S1 before and after adsorption in monocomponent systems for both pollutants. From EDS analysis, a wide distribution of S (which is present in MB molecule) and Cd can be observed on the adsorbent surface, indicating that the adsorption of the dye and the metal ion took place in monocomponent systems. Other metals like K, Ca, Na and Mg were also observed in the elemental analysis by EDS.

Sample: OP+Cd

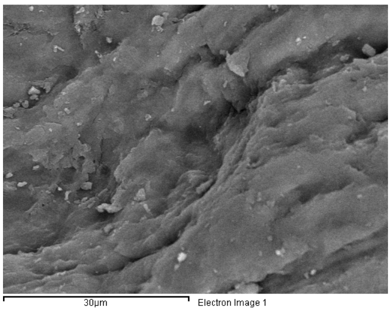
30µm
Electron Image 1

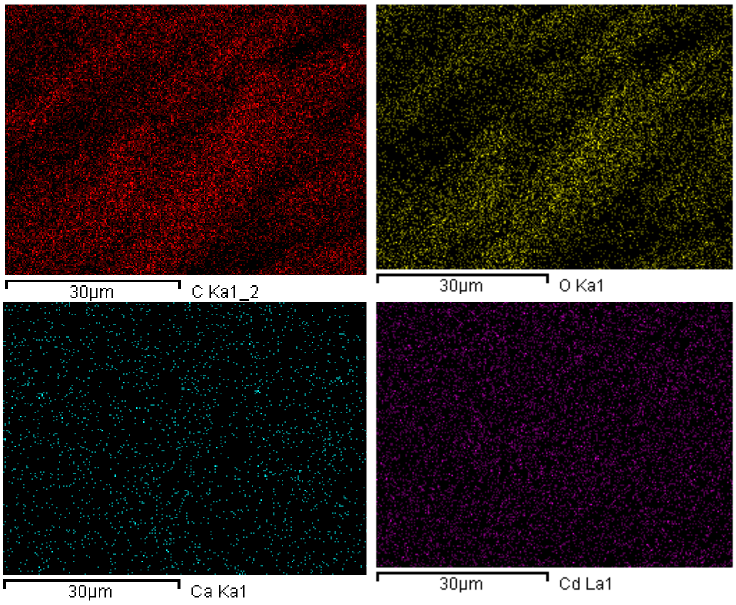
30µm
C Ka1_2
30µm
O Ka1
30µm
Ca Ka1
30µm
Cd La1

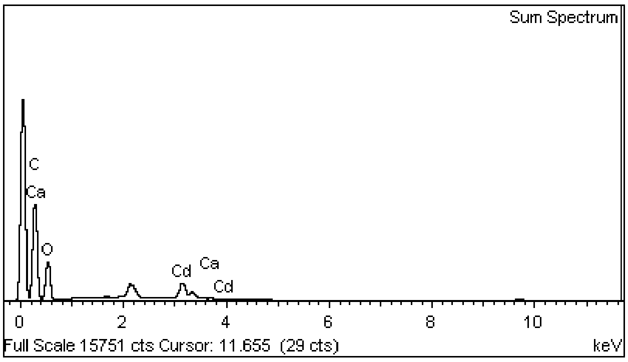
Sum Spectrum
C
Ca
O
Cd
Ca
Cd
0
2
4
6
8
10
Full Scale 15751 cts Cursor: 11.655 (29 cts)
keV

Sample: OP + MB

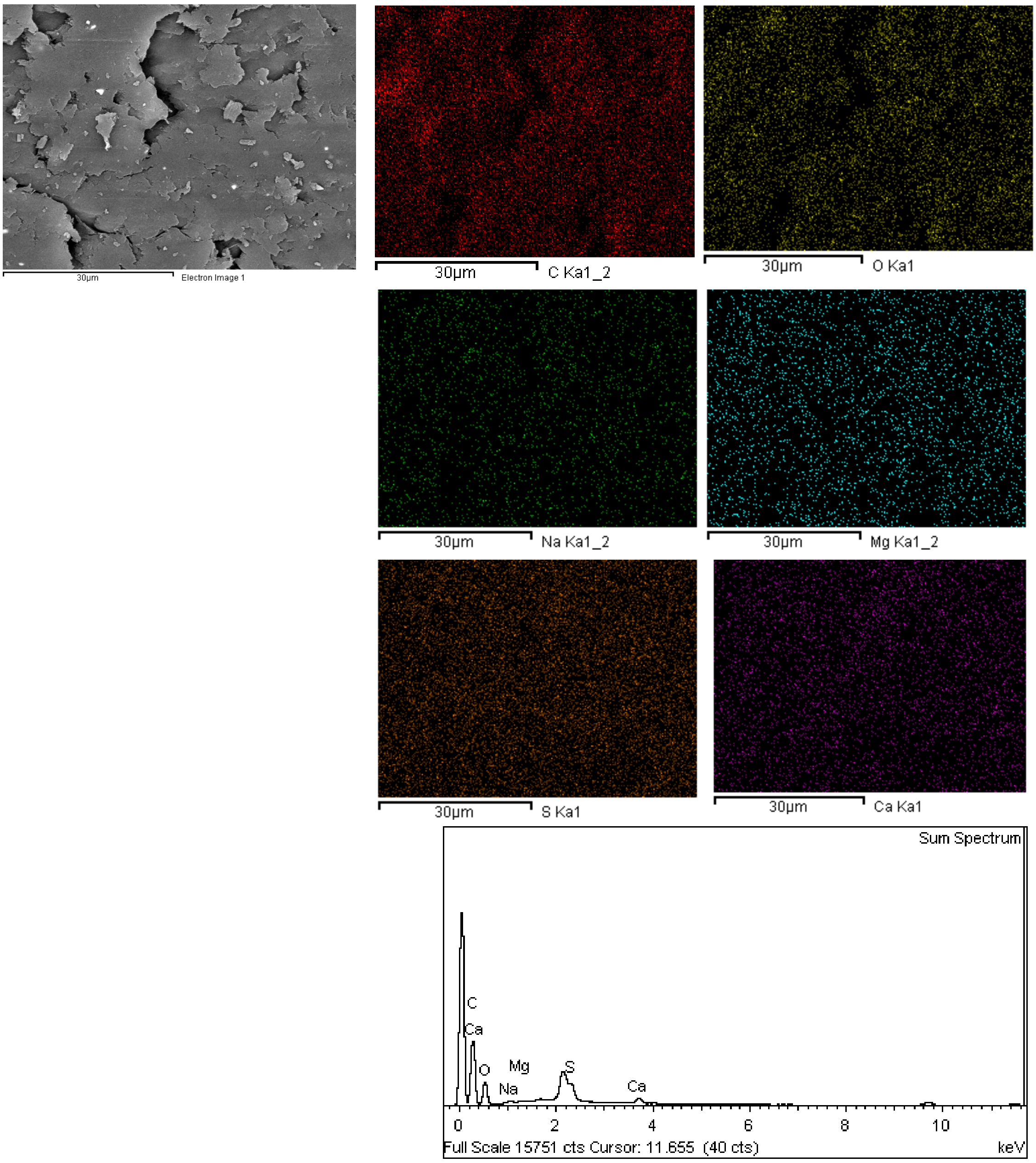
30µm
Electron Image 1
30µm
C Ka1_2
30µm
O Ka1
30µm
Na Ka1_2
30µm
Mg Ka1_2
30µm
S Ka1
30µm
Ca Ka1
Sum Spectrum
C
Ca
O
Mg
Na
S
Ca
0
2
4
6
8
10
Full Scale 15751 cts Cursor: 11.655 (40 cts)
keV

Sample: OP

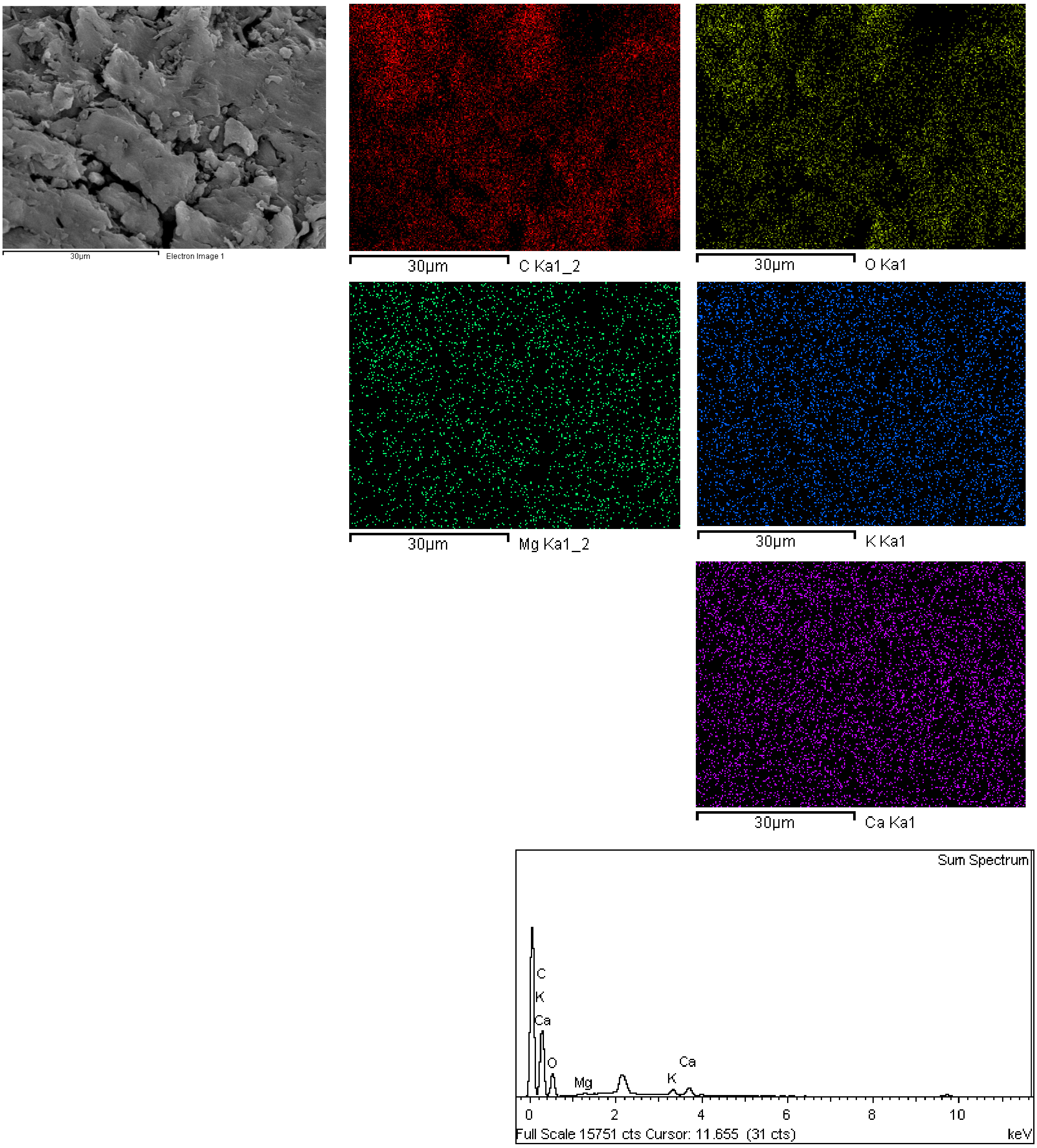

Figure S1. SEM-EDS elemental mapping of OP after MB and Cd^{2+} adsorption in monocomponent systems.
